# Human sex development: targeted technologies to improve diagnosis

**DOI:** 10.1186/s13059-016-1128-4

**Published:** 2016-12-15

**Authors:** Federica Buonocore, John C. Achermann

**Affiliations:** Genetics and Genomic Medicine, UCL Great Ormond Street Institute for Child Health, 30 Guilford Street, London, WC1N 1EH UK

**Keywords:** Disorders of sex development, DSD, Haloplex, Gonadal dysgenesis, Genetic testing, Sex determination

## Abstract

A new study of disorders of sex development presents an improved targeted next-generation sequencing approach for their diagnosis.

Please see related Research article: http://genomebiology.biomedcentral.com/articles/10.1186/s13059-016-1105-y.

## Human sex development

Disorders of (or differences in) sex development (DSD) are a broad range of conditions that can affect reproductive development and function in humans. Typically, a child might first present with atypical genitalia at birth, in such a way that it is not immediately possible to say whether the newborn is a boy or girl. Other individuals can present in childhood or teenage years, or even first in adulthood. It is estimated that approximately 1 in every 4000 people has DSD, although other variations of these conditions (e.g. some forms of hypospadias) are much more prevalent.

**Fig. 1:**
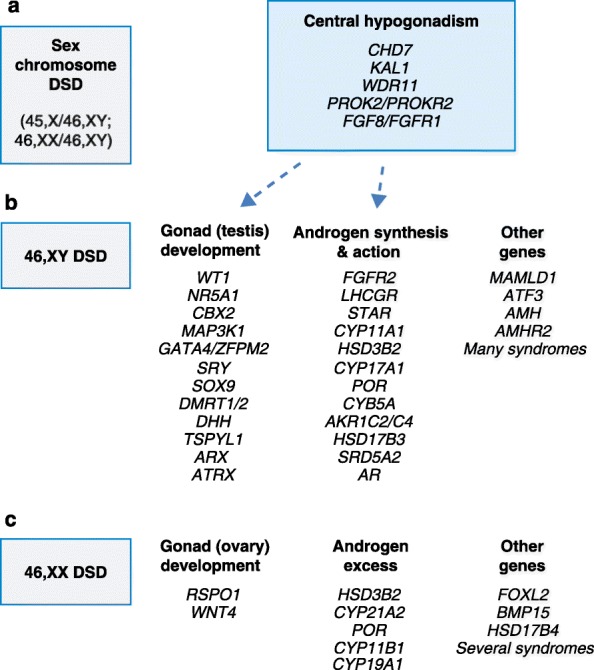
**a**–**c** Overview of some of the single genes currently associated with disorders/differences of sex development (*DSD*). Targeted next-generation sequencing (NGS) panels can be valuable for the parallel analysis of many genes simultaneously, especially for forms of 46,XY DSD (**b**) where the diagnosis is unclear. A study by Eggers and colleagues [5] raises the possibility that variants in genes traditionally associated with central hypogonadism/Kallmann syndrome might also contribute to a 46,XY DSD phenotype

The past 25 years have seen significant progress in our understanding of the genetic basis of sex development and related conditions. In the early 1990s, SRY (‘sex-determining region Y protein’) was identified as the main Y-chromosomal driver for testis development, supported by the generation of ‘Randy’, an XX mouse that developed testes and a male phenotype owing to the presence of a *Sry* transgene [1]. SRY is also crucially important in human testis determination, although only approximately 5% of individuals with testicular dysgenesis have disruption of this transcription factor. More than 40 other genes have now been implicated in human DSD, some of which can disrupt the male-typical development pathway in individuals with a 46,XY karyotype, whereas others can cause excess androgen production in the developing 46,XX child (Fig. 1). Sometimes biochemical data, associated features or family history can help target a diagnosis, especially in conditions affecting steroidogenesis, but, for most children with gonadal dysgenesis or hypospadias, a genetic cause is not typically found [2].

## Moving into the next generation

Making a genetic diagnosis of DSD can have important implications for counselling young people and their families about the likely course of their condition, associated features that might need monitoring, hormonal function and fertility, and gonadal tumor risk [2]. A specific genetic diagnosis can also predict the likelihood of other family members being affected. Traditionally, potential candidate genes have been sequenced one at a time, often as part of research studies. With the exception of changes in steroidogenic factor-1 (NR5A1), the returns are usually meagre [3]. This is in part due to the diverse number of different genes that can cause DSD in affected individuals, each of which only contributes a small percentage to the overall picture.

In recent years, next-generation sequencing (NGS) approaches have provided great potential for upscaling genetic analysis and investigating many genes in parallel. Studies using whole-exome sequencing (WES) have investigated small cohorts of children with DSD and have identified changes in genes known to be altered in DSD as well as novel genes [4]. However, WES approaches have the disadvantage of still being relatively expensive and also producing extensive additional (often unrelated) genetic information of uncertain importance.

An alternative approach, reported in a recent study by Eggers and colleagues, is to use targeted panels of known (and candidate) genes for high-throughput deep sequencing to try to reach a diagnosis for DSD [5]. This involved using a targeted panel (HaloPlex) that included 64 known DSD-associated genes (and 967 candidate genes), in an international multi-centre study of 326 individuals and 129 of their family members. This approach involved many more individuals than previous reports, as well as a wider range of genes and greater depth of sequencing [6, 7]. The panel also included many genes involved in central hypogonadism (central hypogonadotropic hypogonadism, CHH).

The study by Eggers et al. has generated several important findings [5]. First, a likely specific genetic diagnosis was reached in 43% of the individuals tested. Although functional studies were not performed, the American College of Medical Genetic and Genomics guidelines were used to determine the likely pathogenicity of the variants [8]. Second, it was surprising that the analysis of trios (proband and parents) did not significantly improve the diagnostic yield. As many genetic variants associated with DSD arise as de novo dominant conditions, it would have been anticipated that trio analysis would have helped to identify de novo changes and thereby strengthen evidence for likely pathogenicity. Third, 13 individuals with 46,XY DSD had more than one curated variant in a diagnostic DSD gene detected, suggesting a potential oligogenic inheritance in some situations. Finally, there was an enrichment of likely pathogenic variants found in several genes (e.g. *CHD7*, *WDR11*, *FGF8*/*FGFR1*) that are traditionally associated with CHH/Kallmann syndrome. CHH is a hypothalamic–pituitary condition in which release of the gonad-stimulating hormones follicle-stimulating hormone (FSH) and luteinizing hormone (LH) is reduced. In the current model of human sex development, congenital gonadotropin insufficiency should only affect penile growth (length) and testis descent in the later stages of fetal development. The identification of changes in these genes in individuals with hypospadias and other more marked variations in genital anatomy suggest that some genes function at multiple levels throughout the hypothalamic–pituitary–gonadal axis and support previous findings by Baxter and colleagues [4].

Several limitations of this study must also be highlighted. With more-detailed clinical and biochemical phenotyping, it is possible that a diagnosis could still have been reached in a very small number of children using single-gene analysis (e.g. *WT1* in renal failure or *STAR* in lipoid congenital adrenal hyperplasia) [2]. Furthermore, analysis of several classic congenital adrenal hyperplasia (CAH) genes (e.g. *CYP21A2*) can be difficult using targeted capture and NGS because of the presence of a pseudogene. As CAH is a common and important diagnosis to make, a combination of biochemical analysis followed by single-gene testing is still the best approach, and accordingly children with CAH were not entered into this study [2]. Finally, different personal and cultural views on genetic testing need to be considered. Unlocking genetic knowledge is often of benefit, but can also carry a burden of information, especially in such a sensitive area as reproductive development. Engagement and education of families and young people, together with balanced information and consent, are important. While bearing all these considerations in mind, offering a more comprehensive panel-based approach to genetic testing, as described by Eggers and colleagues, certainly seems one way forward.

## Where do we go from here?

High-throughput panel-based genetic analysis has now come of age in many settings, and similar studies have recently been reported for other endocrine conditions. For example, De Franco and colleagues used a targeted NGS panel as part of their comprehensive evaluation of an international cohort of more than 1000 children with neonatal diabetes, where a final molecular diagnosis was reached in 82% [9]. Similarly, Guran and collaborators studied almost 100 patients with primary adrenal insufficiency of unknown cause from a national cohort study in Turkey and reached a genetic diagnosis in 81% [9, 10].

Why then is the diagnostic yield in DSD not more than 50%? Several reasons could account for this:

1. Many of the children included had undergone prior analysis of single genes, and so the diagnostic yield of the array would likely have been higher if the children had been recruited without any prior testing.
2. Key novel genes involved in DSD might yet be discovered. For example, important variants could have been detected in some of the many candidate genes included in the study by Eggers et al. but not included in their report; novel variants might be found in WES studies of DSD currently under way; and genomic changes in enhancers or regulatory regions might be detected by whole-genome sequencing approaches, such as the 100,000 genomes project. International collaborations might be needed to understand the potential contribution of rare variants in diverse genes of unknown function, or to piece together any potential role for complex digenic or oligogenic interactions. Data analysis in DSD can be complicated further by the relative lack of large pedigrees as these genetic conditions often result in infertility and are not transmitted; by the observation of sex-limited inheritance patterns (e.g. where mothers can carry an autosomal dominant variant but are not affected); and by variable phenotypic penetrance. Even where there is a family history, this might be private information that is not widely shared among family members.
3. Several studies have shown that copy-number variants can be involved in DSD, which are not so easily detected using current NGS technologies or bioinformatic pipelines.
4. Somatic changes in key factors during early embryonic life can affect organ development in a tissue- and time-specific manner. Such events would not be detected unless the specific tissues were analysed (e.g. gonad), and this might be impossible once an organ has undergone fibrosis or has regressed.
5. Epigenetic or environmental factors can influence early gonad development or genital anatomy either alone or in combination with rare genetic events.

Despite these many challenges with DSD, human genetics has entered an exciting era, and the study by Eggers and colleagues shows that the field is making progress. Translation of research approaches into clinical service is an important short-term goal, and providing benefits for young people and their family must remain very much the focus of investigators’ work. Smaller-scale targeted NGS panels are already available in clinical service in some centres, with the main drawback being the need to batch several samples together for analysis, which can slow down turnaround times. In the future, larger multi-disorder panels, whole-exome and even whole-genome approaches might be used as first-line investigations, with the initial bioinformatics analysis restricted only to DSD-related genes. In most places, however, these approaches are still limited by cost and capacity, and so in the meantime specific targeted panels have a lot to offer.

## Abbreviations

CAH: Congenital adrenal hyperplasia
CHH: Central hypogonadotropic hypogonadism
DSD: Disorders/differences of sex development
FSH: Follicle-stimulating hormone
LH: Luteinizing hormone
NGS: Next-generation sequencing
WES: Whole-exome sequencing
